# The Looking Glass for Intelligence Quotient Tests: The Interplay of Motivation, Cognitive Functioning, and Affect

**DOI:** 10.3389/fpsyg.2019.02857

**Published:** 2019-12-17

**Authors:** Venkat Ram Reddy Ganuthula, Shuchi Sinha

**Affiliations:** Department of Management Studies, Indian Institute of Technology Delhi, New Delhi, India

**Keywords:** affect, cognitive functioning, intelligence quotient, motivation, intelligence

## Abstract

The Intelligence Quotient (IQ) tests and the corresponding psychometric explanations dominate both the scientific and popular views about human intelligence. Though the IQ tests have been in currency for long, there exists a gap in what they are believed to measure and what they do. While the IQ tests index the quality of cognitive functioning in selected domains of mental repertoire, the applied settings often inflate their predictive value leading to an interpretive gap. The present article contends that studying the influence of motivational and affective processes on cognitive functioning would help to evolve a more psychologically comprehensive account of the IQ tests and bridge the interpretive gap. To conclude, the article suggests possible future research directions that could strengthen the predictive value of the IQ tests.

## Introduction

The need to distinguish ourselves from others around us is perhaps distinctly human. Intelligence as a concept not only sets us apart as a species from the rest of the animals but also enables us to place ourselves uniquely in the company of fellow human beings (Sternberg, 2018). Roughly speaking, the concept of intelligence accounts for the efficacy of the mental functioning that underlies behavior based on specific criteria (Perkins, 1995). However, the set of rules that qualify mental functioning has been a subject of considerable debate between the narrow vs. the broad theories of intelligence (Stanovich, 2009).

The narrow theories identify intelligence with performance on a set of tests that account for cognitive functioning in selected domains of human mental repertoire. The composite of such tests is known as the Intelligence Quotient (IQ) test. Binet and Simon (1916) were among the first to develop the influential tradition of IQ testing. Their objective was to identify cognitively challenged children registered in the French public school system and educate them. To this end, they designed tests that measured how a child’s cognitive functioning shaped mental abilities such as judgment, comprehension, and reasoning. Lewis Terman took this test to Stanford University and revised it to what has come to be known as the Stanford-Binet IQ test (Roid and Pomplun, 2012). From then on, the test has been revised frequently and continues to be used in countries all over the world as a measure of intelligence (Deary, 2001). Many other IQ styled tests, such as the SAT (Scholastic Aptitude Test), have come in vogue with time (Sternberg, 2006). The variety of IQ tests in use today differ in the number and kind of mental abilities they attempt to measure (Sternberg, 2018).

For instance, the one widely used IQ test is the Wechsler Adult Intelligence Scale III (WAIS-III) (The Psychological Corporation, 1997; Wechsler, 1997). WAIS-III measures the performance of an individual on a set of four mental abilities: verbal comprehension, processing speed, perceptual organization, and working memory (Wechsler, 1997). A collection of 13 distinct tasks account for each of the four mental abilities. These tasks, in turn, have a specific number of items that contribute to the overall score.

Psychometric theories characterize the performance on the IQ tests through factor analytic procedures (Deary, 2001; Sternberg, 2006). Typically, a psychometric theory accounts for the performance on the IQ tests in two related steps. Firstly, the performance on the items across a set of related tasks converges to a particular mental ability (Kline, 2013). Then the common variance underlying scores on candidate’s mental abilities converge to a single factor termed as the g-factor. The g-factor so arrived is representative of an individual’s general intelligence (Kline, 2013). The intuitive idea here is that performance across individual mental ability tests (termed as factors) is positively correlated – a phenomenon referred to as “positive manifold” in psychometric parlance (Sternberg, 2018). However, few variants follow a single-step procedure to account for the common variance across the scores on different tasks of the IQ test to arrive at the g-factor (Deary, 2001). Despite the procedural nuances, all the psychometric theories agree that the g-factor culled out of the performance on the IQ tests represents an individual’s intelligence (Eysenck, 2018). Together, the IQ tests and the corresponding psychometric explanations paved the way for the birth of differential psychology – a systematic study of how and why our minds work differently (Eysenck, 2018).

While the narrow theories dominate the scientific and common-sense notion of intelligence, they are not devoid of criticism. Notably, the critique contends that narrow theories are not representative of mental functioning. The IQ tests characterize the efficiency with which an individual gathers and processes information in particular domains that are primarily cognitive. They leave out non-cognitive aspects of mental functioning such as socio-emotional skills and interpersonal capabilities, among others (Neisser et al., 1996). Furthermore, the research raises questions around the representativeness of the tests. Researchers find the IQ tests inadequate in accounting for performance on even cognitively loaded aspects of an individual’s mental life. Related evidence suggests that IQ scores are inconsistent at predicting variation in performance (including the extremities) on activities such as learning, reasoning, and decision-making (Stanovich, 2009; Fletcher et al., 2018).

This critique of the IQ tests leads to the rise of the broad theories of intelligence. The broad theories emphasize on the aspects of mental functioning associated with the vernacular use of the term intelligence including adaptation to the environment, display of wisdom, creativity, etc., irrespective of whether these aspects are amenable to measurement or not (Gardner, 1993; Perkins et al., 1993; Ceci, 1996; Sternberg, 2018). They highlight aspects of mental functioning that shape human behavior that is otherwise largely ignored by the narrow theorists, including biological characteristics, psychological processes, and socio-cultural environs.

However, the narrow theorists accuse the broad perspectives of deliberately expanding the conceptual scope and usage of the term intelligence to counter the elevated status of the IQ tests. They argue that the generous conceptualizations of broad theories strategically downplay the importance of the IQ tests by broadening the definition of intelligence to make them only a part of the larger whole (Stanovich, 2009). Moreover, critics also highlight the fact that broad theories lack empirical grounding and exclusively rely on anecdotal evidence for support (Stanovich et al., 2016). Overall, both the narrow and broad theories form the core of the longstanding debate regarding the nature of intelligence and its measurement. Nevertheless, the narrow approaches with the IQ tests at heart enjoy an excellent scientific and popular reputation in comparison to the broad theories. EG Boring’s (1923) famous assertion that “Intelligence is what IQ tests measure” reflects the status enjoyed by the IQ tests in the scientific community.

## The Interpretive Gap

The use of IQ tests permeates many spheres of human activity (Sternberg et al., 2001). The IQ tests are used to make decisions in a variety of contexts, including school and college admissions, employment opportunities, and even mate selection (Hunt, 1995; Fitzsimons, 2015). The wide-ranging applications of the IQ tests, however, raise specific concerns. Conceptually, the IQ tests index the quality of cognitive functioning in selected aspects of an individual’s mental life.

However, their real-world interpretations inflate them to represent overall mental functioning across wide-ranging domains, from education achievement to job performance and interpersonal relationships (Sternberg et al., 2001). This inflation is evident from the contradictions observed in the evidence on the predictive value of the IQ tests. Related research suggests a weak to moderate correlation between performance on IQ tests and outcomes on education, job performance, income levels, and overall individual well-being (Bowles and Gintis, 2002; Strenze, 2007). Other factors, such as parent’s socio-economic status (Strenze, 2007), have been shown to moderate these correlations actively. Overall, the evidence points to an interpretive gap between what the IQ tests are believed to measure and what they do.

The interpretive gap adversely impacts critical factors that shape human development. Modern meritocratic societies restrict access to opportunities to education, employment, and overall growth to those who fare well on the IQ tests while excluding others who do not do well on them (Neisser et al., 1996). Much of the criticism on the IQ tests mounted by the broad theorists also stems from this interpretive gap (Sternberg, 2018). Therefore, the debate on the nature of intelligence needs to be reframed to address the issues concerning the interpretation of the IQ scores, rather than altogether abandoning these tests.

In this regard, the advances in how motivational and affective processes influence cognitive functioning hold promise. Mental functioning largely rests on the three psychological processes of motivation, cognitive functioning, and affect (Crocker et al., 2013; Pessoa, 2013). Motivational and affective processes shape and reshape cognitive functioning, giving rise to much of the behavioral diversity observed in the real world (Simon, 1967; Crocker et al., 2013).

This article attempts to summarize the evidence on how motivational and affective processes account for cognitive functioning in general and the IQ tests in particular. To conclude, the article lays out specific future research directions. The next sections lay out the role of motivation in different aspects of cognitive functioning and suggest how affect modulates motivations and cognitive functioning.

## Influence of Motivation and Affect on Cognitive Functioning

A salient feature of human behavior is that it is not only organized but also purposive (Ryan, 2012). It is the motivations that imbue an individual’s action with structure and purpose. Motivations are value-laden cues that are an outcome of person-environment interaction (Braver et al., 2014). They carry forward two functions: energization and direction (Heckhausen and Heckhausen, 2018). While energization instigates or activates the individual’s cognitive functioning, the direction function orients the energized cognitive repertoire to specific ends (Elliot, 2008). Evidence compiled over decades of research indicates that motivations influence various aspects of cognitive functioning ranging from rudimentary perception (Rothkirch and Sterzer, 2015), to more complex attention (Rothkirch et al., 2014), learning (Daw and Shohamy, 2008), memory (Miendlarzewska et al., 2016), and control (Botvinick and Braver, 2015).

Motivations drive expectations that bias human vision and perceptual mechanisms to selectively process features of the visual environment (O’Callaghan et al., 2017). This skewing of the perceptual apparatus impacts the estimates of size, distance, steepness, and salience of the objects in the visual environment (Firestone and Scholl, 2016). Moreover, expectations also help interpret ambiguous stimuli and make meaning of the perceptual settings even when constrained on information (O’Callaghan et al., 2017). Likewise, motivations also drive higher level information search and processing underlying reasoning, judgment, and decision-making (Chiew and Braver, 2011; Epley and Gilovich, 2016). They orient attention mechanisms to selectively acquire information and modulate parameters such as speed, accuracy, and depth of information processing (Dweck et al., 2004).

Further, the motivational cues also drive learning mechanisms ranging from simple associative to more complex conditioning strategies that help to establish relationships between distinct pieces of information (Dayan and Balleine, 2002; Daw and Shohamy, 2008). They importantly modulate the strength of the learning (Braver et al., 2014). Relatedly, research also suggests that motivational relevance modulates encoding and retrieval of acquired information (Miendlarzewska et al., 2016).

Motivations also facilitate control processes that help choose between competing motivations (Botvinick and Braver, 2015; Suri et al., 2018). This preferential treatment of some motivations over others enables not only cognitive functioning to swiftly shift from one information environment to the other (Suri et al., 2018), but also drive behavioral responses within the selected context (Yee and Braver, 2018).

However, the successful pursuit of motivation also requires continuous monitoring and feedback (Carver, 2018). Monitoring enables people to be alerted to the congruence between the current behavior and its consequences to the characteristics of the desired actions and outcomes (Benn et al., 2014). This continuous check over motivation referent behaviors warrants people to identify the discrepancies and close the gaps between the current and desired behavioral responses (Harkin et al., 2016).

Feedback from periodic monitoring of the motivation referent behavior takes the shape of affect (Fishbach and Finkelstein, 2012). The positive affective states (i.e., good-for-me feelings) convey advancements in motivational pursuits, while the negative affective states (i.e., bad-for-me feelings) signal discrepancies in purposive behavior (Hart and Gable, 2013; Inzlicht et al., 2015). Further, the positive affect strengthens motivational intensity (Orehek et al., 2011), while the negative affect typically weakens it (Watkins and Moberly, 2009). These changes to motivations because of affect impact subsequent cognitive functioning and behavior (Carver and Scheier, 2008; Gable and Harmon-Jones, 2010; Gable et al., 2016).

Recent neuroscientific evidence also supports the interplay between the three strands of mental functioning (Pessoa, 2019). Several anatomical and functional studies suggest that brain regions are highly interconnected. These interconnected networks form the basis of interaction among motivation, cognitive functioning, and affective processes (Pessoa, 2013). In all, motivational and affective processes influence cognitive functioning significantly. This evidence has implications for the IQ tests and their interpretation.

## Role of Motivation and Affect on Performance in the Intelligence Quotient Tests

Growing evidence suggests that motivations energize and guide the cognitive performance of a typical test taker (Duckworth et al., 2011). Relatedly, the dispositional theory of intelligence (Perkins et al., 1993) predicts that trait motivations drive much of the variation in performance on the IQ tests. Likewise, research suggests that traits such as growth mindset, openness to experience, and need for cognition modulate the willingness to search and process information that, in turn, influences an individual’s performance on an IQ test (Dweck, 2006; Woods et al., 2019).

However, a recent meta-analytic review of motivational influences on cognitive performance suggests that dispositional traits account for less considerable variation when compared to shifts in motivational states (Van Iddekinge et al., 2018). In a seminal study, Duckworth et al. (2011) present evidence on how state changes in test taker’s motivations significantly predict performance on the IQ tests. The research also suggests that the predictive validity of the IQ scores for various life outcomes substantially diminishes with the shifts in motivational levels of the test taker.

Feedback on performance and subsequent affective states influence the cognitive functioning of the test taker. Mainly, the negative affective states like task anxiety have been found to lower the performance on the IQ tests substantially (von der Embse et al., 2018). To sum up, sparse but significant evidence on motivational and affective processes suggests that they account for substantial variation in performance on the IQ tests.

## Implications and Future Research Directions

The evidence on the impact of motivational and affective processes has implications for the interpretation and use of IQ tests. Conventionally, differences in performance on the IQ tests have been assumed to solely convey discrepancies in the quality of cognitive functioning of the test takers. However, with the new evidence on the anvil, variability in performance on the IQ tests also appears to be a function of the kind and intensity of motivations and affective states the test takers experience during the test. This evidence calls for a change in the way the IQ scores are interpreted to make real-world decisions. Therefore, moving forward, more concerted efforts at unearthing the effects of motivational and affective processes on cognitive functioning in the context of the IQ tests are necessary.

Notably, future research could examine what kind of motivational cues, i.e., task vs. outcome-oriented (Pintrich, 2000), are optimum for performance on an IQ test. An individual with task-oriented motivation perceives doing well on an IQ test as an end in itself. In contrast, the individual driven by outcome-oriented motivation assumes performing on an IQ test as instrumental to other life outcomes. Further, research needs to examine how motivational intensity (whether task-oriented or outcome-oriented) modulates cognitive functioning on an IQ test.

Research on goal-directed behaviors suggests that monitoring and feedback might as well account for variation in cognitive functioning (Fishbach et al., 2010; Carver, 2018). Therefore, future studies could also examine how the frequency of monitoring and the nature of feedback influence performance on IQ tests. Likewise, affective responses to monitoring performance during the IQ test could also account for the overall performance on the test. Pertinent research only examined the impact of negative affective states such as anxiety (von der Embse et al., 2018). However, positive affective states could also contribute to the variation in IQ scores (Fredrickson, 2004). Lastly, studies could also examine how trait emotion regulation strategies influence performance on IQ tests as they modulate the generation and expression of emotions (Gross, 2002).

Put together, these strands of research could eventually contribute to a more psychologically nuanced account of the IQ tests. Such an integrated view would help to purge the interpretive gap that plagues their real-world applications.

## Conclusion

The notion of intelligence is here to stay, and so are the IQ tests that index intelligence. However, there is a case for a more psychologically comprehensive interpretation of what IQ scores reflect. Examining the influence of motivational and affective processes on cognitive functioning underlying performance on the IQ tests is a step in this direction. A reliable account of what the IQ scores reflect would enable a more cautioned use of these numbers to determine access to opportunities that shape individual life outcomes in modern meritocratic societies.

## Author Contributions

Both VG and SS contributed equally at all stages of the development of the manuscript leading to its submission.

### Conflict of Interest

The authors declare that the research was conducted in the absence of any commercial or financial relationships that could be construed as a potential conflict of interest.

## References

[ref1] Benn Y. Webb T. L. Chang B. P. Sun Y. H. Wilkinson I. D. Farrow T. F. (2014). The neural basis of monitoring goal progress. Front. Hum. Neurosci. 8:688. 10.3389/fnhum.2014.00688, PMID: 25309380 PMC4159987

[ref2] Binet A. Simon T. (1916). “The development of intelligence in children” in *The Binet-Simon scale*. trans. Kite E. S. (Baltimore, MD: Williams & Wilkins Co.).

[ref3] Boring E. G. (1923). Intelligence as the tests test it. *New Republic*. 35–37.

[ref4] Botvinick M. Braver T. (2015). Motivation and cognitive control: from behavior to neural mechanism. Annu. Rev. Psychol. 66, 83–113. 10.1146/annurev-psych-010814-015044, PMID: 25251491

[ref5] Bowles S. Gintis H. (2002). The inheritance of inequality. J. Econ. Perspect. 16, 3–30. 10.1257/089533002760278686

[ref6] Braver T. S. Krug M. K. Chiew K. S. Kool W. Westbrook J. A. Clement N. J.. (2014). Mechanisms of motivation-cognition interaction: challenges and opportunities. Cogn. Affect. Behav. Neurosci. 14, 443–472. 10.3758/s13415-014-0300-0, PMID: 24920442 PMC4986920

[ref7] Carver C. S. (2018). Control theory, goal attainment, and psychopathology. Psychol. Inq. 29, 139–144. 10.1080/1047840X.2018.1513681, PMID: 31447528 PMC6707771

[ref8] Carver C. S. Scheier M. F. (2008). On the self-regulation of behavior. Cambridge, UK: Cambridge University Press.

[ref9] Ceci S. J. (1996). On intelligence: A bio-ecological treatise on the development of intelligence. Cambridge, MA: Harvard University Press.

[ref10] Chiew K. S. Braver T. S. (2011). Positive affect versus reward: emotional and motivational influences on cognitive control. Front. Psychol. 2:279. 10.3389/fpsyg.2011.00279, PMID: 22022318 PMC3196882

[ref11] Crocker L. D. Heller W. Warren S. L. O'Hare A. J. Infantolino Z. P. Miller G. A. (2013). Relationships among cognition, emotion, and motivation: implications for intervention and neuroplasticity in psychopathology. Front. Hum. Neurosci. 7:261. 10.3389/fnhum.2013.00261, PMID: 23781184 PMC3678097

[ref12] Daw N. D. Shohamy D. (2008). The cognitive neuroscience of motivation and learning. Soc. Cogn. 26, 593–620. 10.1521/soco.2008.26.5.593

[ref600] Dayan P. Balleine B. W. (2002). Reward, motivation, and reinforcement learning. Neuron 36, 285–298. 10.1016/s0896-6273(02)00963-7 12383782

[ref13] Deary I. J. (2001). Intelligence: A very short introduction. Oxford, UK: Oxford University Press.

[ref14] Duckworth A. L. Quinn P. D. Lynam D. R. Loeber R. Stouthamer-Loeber M. (2011). Role of test motivation in intelligence testing. Proc. Natl. Acad. Sci. USA 108, 7716–7720. 10.1073/pnas.1018601108, PMID: 21518867 PMC3093513

[ref15] Dweck C. S. (2006). Mindset: The new psychology of success. New York, NY: Random House.

[ref16] Dweck C. S. Mangels J. A. Good C. (2004). “Motivational effects on attention, cognition, and performance” in The educational psychology series. Motivation, emotion, and cognition: Integrative perspectives on intellectual functioning and development. eds. Dai D. Y. Sternberg R. J. (Mahwah, NJ: Lawrence Erlbaum Associates Publishers), 41–55.

[ref17] Elliot A. J. (Ed.) (2008). Handbook of approach and avoidance motivation. New York, NY: Psychology Press.

[ref18] Epley N. Gilovich T. (2016). The mechanics of motivated reasoning. J. Econ. Perspect. 30, 133–140. 10.1257/jep.30.3.133

[ref19] Eysenck H. J. (2018). Intelligence: A new look. Piscataway, NJ: Transaction Publishers.

[ref20] Firestone C. Scholl B. J. (2016). Cognition does not affect perception: evaluating the evidence for “top-down” effects. Behav. Brain Sci. 39:e229. 10.1017/S0140525X15000965 26189677

[ref21] Fishbach A. Eyal T. Finkelstein S. R. (2010). How positive and negative feedback motivate goal pursuit. Soc. Pers. Psychol. Compass 4, 517–530. 10.1111/j.1751-9004.2010.00285.x

[ref22] Fishbach A. Finkelstein S. R. (2012). “How feedback influences persistence, disengagement, and change in goal pursuit” in Goal-directed behavior. eds. Aarts H. Elliot A. J. (London, UK: Psychology Press), 203–230.

[ref23] Fitzsimons P. (2015). “Human capital theory and education” in Encyclopedia of educational philosophy and theory. ed. Peters M. A. (Singapore: Springer Publishing), 1–4.

[ref24] Fletcher J. M. Lyon G. R. Fuchs L. S. Barnes M. A. (2018). Learning disabilities: From identification to intervention. New York, NY: The Guilford Press.

[ref25] Fredrickson B. L. (2004). The broaden-and-build theory of positive emotions. Philos. Trans. R. Soc. Lond. Ser. B Biol. Sci. 359, 1367–1377. 10.1098/rstb.2004.1512, PMID: 15347528 PMC1693418

[ref26] Gable P. A. Browning L. Harmon-Jones E. (2016). “Affect, motivation, and cognitive scope” in Frontiers of cognitive psychology. Motivation and cognitive control. ed. Braver T. S. (New York, NY: Routledge/Taylor & Francis Group), 164–187.

[ref27] Gable P. Harmon-Jones E. (2010). The motivational dimensional model of affect: implications for breadth of attention, memory, and cognitive categorisation. Cognit. Emot. 24, 322–337. 10.1080/02699930903378305

[ref28] Gardner H. (1993). Multiple intelligences: The theory in practice. New York, NY: Basic Books.

[ref29] Gross J. J. (2002). Emotion regulation: affective, cognitive, and social consequences. Psychophysiology 39, 281–291. 10.1017/S0048577201393198, PMID: 12212647

[ref30] Harkin B. Webb T. L. Chang B. P. Prestwich A. Conner M. Kellar I.. (2016). Does monitoring goal progress promote goal attainment? A meta-analysis of the experimental evidence. Psychol. Bull. 142, 198–229. 10.1037/bul0000025, PMID: 26479070

[ref31] Hart W. Gable P. A. (2013). Motivating goal pursuit: the role of affect motivational intensity and activated goals. J. Exp. Soc. Psychol. 49, 922–926. 10.1016/j.jesp.2013.05.002

[ref32] Heckhausen J. Heckhausen H. (eds.) (2018). “Motivation and action: introduction and overview” in Motivation and action (New York, NY: Springer), 1–14.

[ref33] Hunt E. (1995). The role of intelligence in modern society. Am. Sci. 83, 356–369.

[ref34] Inzlicht M. Bartholow B. D. Hirsh J. B. (2015). Emotional foundations of cognitive control. Trends Cogn. Sci. 19, 126–132. 10.1016/j.tics.2015.01.004, PMID: 25659515 PMC4348332

[ref35] Kline P. (2013). Intelligence: The psychometric view. London, UK: Routledge.

[ref36] Miendlarzewska E. A. Bavelier D. Schwartz S. (2016). Influence of reward motivation on human declarative memory. Neurosci. Biobehav. Rev. 61, 156–176. 10.1016/j.neubiorev.2015.11.015, PMID: 26657967

[ref37] Neisser U. Boodoo G. Bouchard T. J. Jr. Boykin A. W. Brody N. Ceci S. J. (1996). Intelligence: knowns and unknowns. Am. Psychol. 51, 77–101. 10.1037/0003-066X.51.2.77

[ref38] O’Callaghan C. Kveraga K. Shine J. M. Adams R. B. Jr. Bar M. (2017). Predictions penetrate perception: converging insights from brain, behaviour and disorder. Conscious. Cogn. 47, 63–74. 10.1016/j.concog.2016.05.003 27222169 PMC5764074

[ref39] Orehek E. Bessarabova E. Chen X. Kruglanski A. W. (2011). Positive affect as informational feedback in goal pursuit. Motiv. Emot. 35, 44–51. 10.1007/s11031-010-9197-2, PMID: 21423342 PMC3045658

[ref40] Perkins D. (1995). Outsmarting IQ: The emerging science of learnable intelligence. New York, NY: Simon and Schuster.

[ref41] Perkins D. N. Jay E. Tishman S. (1993). Beyond abilities: a dispositional theory of thinking. Merrill-Palmer Q. 39, 1–21.

[ref42] Pessoa L. (2013). The cognitive-emotional brain: From interactions to integration. Cambridge, MA: MIT Press.

[ref43] Pessoa L. (2019). Intelligent architectures for robotics: the merging of cognition and emotion. arXiv [Preprint]. Available at: http://arXiv:1902.00363 (Accessed October 23, 2019). 10.1016/j.plrev.2019.04.009 31668845

[ref44] Pintrich P. R. (2000). “The role of goal orientation in self-regulated learning” in Handbook of self-regulation. eds. Boekaerts M. Pintrich P. R. Zeidner M. (San Diego, CA: Academic Press), 451–502.

[ref45] Roid G. H. Pomplun M. (2012). The Stanford-Binet intelligence scales. New York, NY: The Guilford Press.

[ref46] Rothkirch M. Schmack K. Deserno L. Darmohray D. Sterzer P. (2014). Attentional modulation of reward processing in the human brain. Hum. Brain Mapp. 35, 3036–3051. 10.1002/hbm.22383, PMID: 24307490 PMC6869517

[ref47] Rothkirch M. Sterzer P. (2015). “The role of motivation in visual information processing” in Motivation and cognitive control ed. Braver T. S. (London, UK: Routledge), 35–61.

[ref48] Ryan R. M. (Ed.) (2012). The Oxford handbook of human motivation. New York, NY: Oxford University Press.

[ref49] Simon H. A. (1967). Motivational and emotional controls of cognition. Psychol. Rev. 74, 29–39. 10.1037/h0024127, PMID: 5341441

[ref50] Stanovich K. E. (2009). What intelligence tests miss: The psychology of rational thought. New Haven, CT: Yale University Press.

[ref51] Stanovich K. E. West R. F. Toplak M. E. (2016). The rationality quotient: Toward a test of rational thinking. Cambridge, MA: MIT Press.

[ref52] Sternberg R. J. (2006). Intelligence. Encyclopedia of cognitive science. New York, NY: Wiley Publishing.

[ref53] Sternberg R. J. (Ed.) (2018). The nature of human intelligence. Cambridge, UK: Cambridge University Press.

[ref54] Sternberg R. J. Grigorenko E. L. Bundy D. A. (2001). The predictive value of IQ. Merrill-Palmer Q. 47, 1–41. 10.1353/mpq.2001.0005

[ref55] Strenze T. (2007). Intelligence and socioeconomic success: a meta-analytic review of longitudinal research. Intelligence 35, 401–426. 10.1016/j.intell.2006.09.004

[ref56] Suri G. Shine J. M. Gross J. J. (2018). Why do we do what we do? The attention–readiness–motivation framework. Soc. Pers. Psychol. Compass 12:e12382. 10.1111/spc3.12382

[ref57] The Psychological Corporation (1997). WAIS-III-WMS-III technical manual. San Antonio, TX: The Psychological Corporation.

[ref58] Van Iddekinge C. H. Aguinis H. Mackey J. D. DeOrtentiis P. S. (2018). A meta-analysis of the interactive, additive, and relative effects of cognitive ability and motivation on performance. J. Manag. 44, 249–279. 10.1177/0149206317702220

[ref59] von der Embse N. Jester D. Roy D. Post J. (2018). Test anxiety effects, predictors, and correlates: a 30-year meta-analytic review. J. Affect. Disord. 227, 483–493. 10.1016/j.jad.2017.11.048, PMID: 29156362

[ref60] Watkins E. R. Moberly N. J. (2009). Concreteness training reduces dysphoria: a pilot proof-of-principle study. Behav. Res. Ther. 47, 48–53. 10.1016/j.brat.2008.10.014, PMID: 19036353 PMC2807031

[ref61] Wechsler D. (1997). WAIS-III administration and scoring manual. San Antonio, TX: Psychological Corporation.

[ref62] Woods S. A. Hinton D. P. von Stumm S. Bellman-Jeffreys J. (2019). Personality and intelligence: examining the associations of investment-related personality traits with general and specific intelligence. Eur. J. Psychol. Assess. 35, 206–216. 10.1027/1015-5759/a000391

[ref63] Yee D. M. Braver T. S. (2018). Interactions of motivation and cognitive control. Curr. Opin. Behav. Sci. 19, 83–90. 10.1016/j.cobeha.2017.11.009, PMID: 30035206 PMC6051692

